# The effect of operating room nursing intervention on the psychological status and incidence of emergence agitation in the recovery period of general anesthesia: A protocol for systematic review and meta-analysis

**DOI:** 10.1097/MD.0000000000027703

**Published:** 2021-11-12

**Authors:** Hongying Zhu, Liurong Cheng, Ting Tang, Yajuan Ke, Duozhi Wu, Yi Huang

**Affiliations:** a Department of operating room, Hainan General Hospital, Hainan Affiliated Hospital of Hainan Medical University, Haikou, Hainan, China; b Department of Anesthesia, Hainan General Hospital, Hainan Affiliated Hospital of Hainan Medical University, Haikou, Hainan, China.

**Keywords:** anxiety, depression, emergence agitation, meta-analysis, operating room nursing intervention, protocol

## Abstract

**Background:**

During the recovery period of general anesthesia, patients are disturbed by residual anesthetic drugs, manifesting as clinical signs of confusion, drowsiness and disorientation, and even abnormal psychology and limb agitation at varying degrees in severe cases. These stress reactions are detrimental to the postoperative recovery, which can be life-threatening. Operating room nursing intervention (ORNI) is a novel nursing model that prevents stress reactions during the recovery period of general anesthesia. However, whether ORNI can improve the psychological condition and reduce the incidence of emergence agitation in the recovery period of general anesthesia remains controversial. Therefore, this study aims to evaluate the effect of ORNI on the psychological status and incidence of emergence agitation in the recovery period of general anesthesia through a systematic review and meta-analysis, thus providing clinical evidence to support it.

**Methods:**

Randomized controlled trials reporting the effect of ORNI on the recovery period of general anesthesia published before October 2021 will be searched in the Chinese Scientific Journal Database, China National Knowledge Infrastructure Database, Wanfang, China Biomedical Literature Database, PubMed, Embase, the Cochrane Library, and Web of Science. Eligible literatures will be screened out according to inclusion and exclusion criteria, and their quality will be assessed using the Cochrane Risk of Bias Assessment Tool. Meta-analysis will be performed using Revman 5.4 software.

**Results:**

This study will evaluate the effect of the ORNI on the recovery period of general anesthesia by calculating the incidence of emergence agitation, and grading the self-rating anxiety scale and self-rating depression scale scores.

**Conclusion:**

This study will provide a reliable evidence-based basis for the application of ORNI in the recovery period of general anesthesia.

**OSF Registration number:**

DOI 10.17605/OSF.IO/P3A4T.

## 1 Introduction

Compared with local anesthesia, large doses of anesthetics are required in the general anesthesia, which would be residual in the patient's body after general anesthesia. As a result, the consciousness and senses have not yet been recovered during the postoperative awakening stage, which can easily be stimulated by the residual anesthetics and thus causes blurred consciousness, limb agitation and abnormal psychology. Residual anesthetics in the recovery period of general anesthesia greatly reduce the patient's compliance and affects the postoperative recovery.^[1,2]^ Emergence agitation is a combination condition of abnormal excitement, limb agitation and disorientation during the recovery period in which the anesthetized state returns full wakefulness.^[3–5]^ Agitation during the awakening period is mainly associated with inadequate preoperative preparation, abnormal sensory perception and residual anesthetic drugs.^[3,5–17]^ Its symptoms are manifested by limb agitation, poor cooperation and compliance, and reflex confrontation at varying degrees. At this period, patients are unable to control themselves and may even subconsciously pull out various devices and tubes attached to the skin, or unconsciously roll over and struggle, resulting in accidents like falling off of the bed, suffocation, and wound dehiscence.^[18–20]^ Various stress reactions can also lead to abnormal psychological changes, often with adverse emotions like despair, anxiety, and depression^[21,22]^ that tend to prolong the awakening period and increase the incidence of emergence agitation.^[4,5,19,23–25]^

Sedative and analgesic medications are often used for symptomatic management of agitation symptoms during the awakening period of general anesthesia. Agitation symptoms can be gradually relieved, but they also cause a prolonged awakening period. Conventional nursing interventions also provide symptomatic management for appearing agitation. However, they are unable to prevent it, and untimely nursing interventions may cause accidental injuries and unfavorable postoperative recovery. Operating room nursing interventions (ORNIs) can anticipate and effectively avoid potential complications of general anesthesia.^[3,19,23,25–28]^ They are patient-targeted and carried out preoperatively, intraoperatively and postoperatively to reduce the stress response. It is reported that ORNIs have achieved significant results in patients undergoing oncology, thyroid, and laparoscopic surgery.^[6,7,10–13,29–32]^

As the effect of ORNI on the psychological status and incidence of emergence agitation in the recovery period of general anesthesia is unclear, this study aims to evaluate it through systematic review and meta-analysis, thus providing an evidence-based basis for clinical implementation of ORNI.

## 2 Methods

### 2.1 Protocol register

This protocol of systematic review and meta-analysis has been drafted under the guidance of the preferred reporting items for systematic reviews and meta-analyses protocols.^[33]^ Moreover, it has been registered on open science framework on October 2021 (Registration number: DOI 10.17605/OSF.IO/P3A4T).

### 2.2 Ethics

Since the protocol does not require patient recruitment and collection of personal information, it does not require ethics committee approval.

### 2.3 Eligibility criteria of inclusion of studies

#### 2.3.1 Types of studies

All randomized controlled trials (RCTs) reporting the effects of ORNI on the psychological status and incidence of agitation in the recovery period of general anesthesia.

#### 2.3.2 Types of participants

Surgically treated patients over 18 years in general anesthesia.

#### 2.3.3 Types of interventions

(1) Conventional nursing interventions are given in the control group: preoperative preparation and health education before surgery; intraoperative monitoring of vital signs and safety instructions; and regular postoperative rounds and symptomatic care.
(2) ORNIs are given in the observation group: preoperative visits, psychological counseling, individualized health education, and explanation of key points of cooperation; standardized ORNIs intraoperatively with a strict sterile environment, including surgical equipment; controlling the flow of operating room staff; communicating with patients after entering the operating room to psychologically guide patients to overcome their fears, worry and other negative emotions, trust the medical staff, and enhance the confidence in surgical treatment; control the temperature and humidity in the operating room within the appropriate range and make the operating room in a reasonable distribution; and explain possible symptoms to patients after surgery, and corresponding treatment; regular rounds are performed to provide psychological guidance and safety protection like the protective restraint.

#### 2.3.4 Types of outcome measures

Outcomes include the incidence of emergence agitation, and self-rating anxiety scale and self-rating depression scale scores.

### 2.4 Exclusion criteria

(1) Duplicate publications;
(2) RCT with incomplete data;
(3) Studies with inconsistent outcomes;
(4) Animal experiments.

### 2.5 Searching strategy

RCTs reporting the effect of ORNI on the recovery period of general anesthesia published before October 2021 will be searched in the Chinese Scientific Journal Database, China National Knowledge Infrastructure Database, Wanfang, China Biomedical Literature Database, PubMed, Embase, the Cochrane Library, and Web of Science using a combination of MeSH terms and free terms. The detailed search strategy of PubMed was given in Table 1.

**Table 1:** Search strategy of the PubMed.

Number	Search terms
#1	Anesthesia, general[MeSH]
#2	Anesthesias, general[Title/Abstract]
#3	General anesthesia[Title/Abstract]
#4	General anesthesias[Title/Abstract]
#5	OR/1 to 4
#6	Operating room nursing[MeSH]
#7	Operating room nursing intervention[Title/Abstract]
#8	Nursing, operating room[Title/Abstract]
#9	Perioperative nursing[MeSH]
#10	Surgical nursing[Title/Abstract]
#11	Nursing, perioperative[Title/Abstract]
#12	Nursing, surgical[Title/Abstract]
#13	Perianesthesia nursing[Title/Abstract]
#14	Nursing, perianesthesia[Title/Abstract]
#15	OR/6 to 14
#16	Randomized controlled trial[MeSH]
#17	Random∗[Title/Abstract]
#18	Clinic trial [Title/Abstract]
#19	OR/16 to 18
#20	#5 AND #15 AND #19

### 2.6 Data collection and analysis

**Figure 1:**
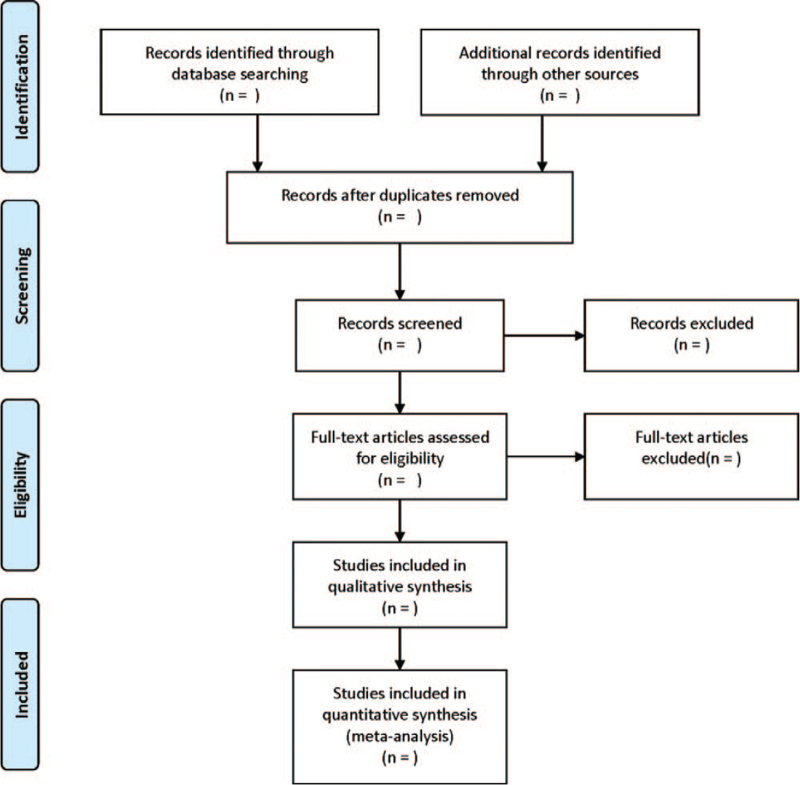
PRISMA flow diagram of the study selection process. PRISMA = preferred reporting items for systematic reviews and meta-analysis.

RCTs will be screened by 2 researchers independently according to the inclusion and exclusion criteria. EndNote will be used for title list management. Duplicate title lists will be first excluded, and the abstract and full text will be further thoroughly reviewed. Disagreement will be resolved through discussing or consulting with the third research. The following data will be extracted: baseline characteristics of the literature: title, first author, year of publication, and source; baseline characteristics of patients: age, gender, disease type, and name of surgery; interventions: intervention details in control and observation groups; evaluation of literature quality according to the Cochrane Risk bias assessment tool and graded into A, B, and C; and outcome indicators: incidence of agitation, self-rating anxiety scale scores, and self-rating depression scale scores. A preferred reporting items for systematic reviews and meta-analysis flow diagram will be used to summarize the results of the whole selection process (Fig. 1).

### 2.7 Literature quality assessment

The quality of the included literature will be evaluated using the Cochrane Risk of Bias Assessment Tool. Risk of bias will be evaluated in 6 aspects, including random allocation methods, allocation concealment, blinding, completeness of outcome indicators, selective reporting of study results, and other sources of bias. Each indicator will be rated as low risk, unclear, or high risk. The results of the risk of bias evaluation will be classified as A (low risk of bias), B (moderate risk of bias), and C (high risk of bias).

### 2.8 Statistical analysis

#### 2.8.1 Data analysis and processing

Meta-analysis will be performed using RevMan 5.4 software. The χ^2^ and I^2^ will be calculated to determine the heterogeneity of recruited literatures. If *P* < .1 and/or I^2^ > 50%, a random-effects model will be used for the combined analysis of heterogeneity; Otherwise, a fixed-effects model will be adopted. Relative risk and standardized mean difference will be assessed as the effect indicator for dichotomous and continuous variables, respectively. Corresponding 95% confidence intervals will be used for all interval estimates.

#### 2.8.2 Management of missing data

Incomplete or nonreported data will be required by contacting the first author via telephone or e-mail. RCTs with unavailable missing data will be excluded.

#### 2.8.3 Subgroup analysis

Subgroup analysis will be performed based on the age and type of disease of the patients.

#### 2.8.4 Sensitivity analysis

To test the stability of the meta-analysis results, we will perform sensitivity analysis using the one-by-one elimination method.

#### 2.8.5 Assessment of publication biases

Funnel plots will be plotted to assess publication biases when less than 10 RCTs are included.

#### 2.8.6 Evidence quality evaluation

The evidence for outcome indicators will be assessed through the grading of recommendation assessment, development and evaluation scoring method,^[34]^ including the risk of bias, indirectness, inconsistency, imprecision, and publication bias. The quality of evidence will be rated as high, moderate, low or very low.

## 3 Discussion

Patients in the recovery period of general anesthesia may experience central nervous system effects at varying degrees due to residual anesthetics.^[5,35,36]^ They are prone to disorientation, limb agitation and abnormal psychology during the awakening period. Combined with the physical weakness of the postoperative patient, postoperative patient agitation and abnormal psychology cause adverse events on the recovery and life safety.^[6,8,19,24,37]^ Conventional nursing interventions in improving abnormal psychological manifestations, sedation and analgesia, and reducing the incidence of emergence agitation in the recovery period of general anesthesia has some limitations and may also accompany an increased risk.

ORNI is a novel nursing model that implements individualized nursing care based on preoperative, intraoperative and postoperative care. Compared with conventional nursing care, ORNI is featured as individualized health education, psychological care, and follow-up care.^[7,26,28]^ Through encouraging, building trust and eliminating adverse emotions, ORNI is capable of reducing the occurrence of agitation, waking up the patient as soon as possible and avoiding the poor outcomes of surgery. At present, clinical studies on ORNI have obtained positive results. However, there is no systematic and comprehensive meta-analysis on the favorable outcomes of ORNI. This systematic review and meta-analysis aims to provide an objective evidence-based basis for the development of ORNI.

Some limitations should be noted: only Chinese and English-published RCTs are recruited, which may have publication bias; only adult patients are recruited, which has limitations in the guidance for children; and only published literatures are recruited, and nonrecruited gray literatures cause potential publication bias.

## Author contributions

**Conceptualization:** Hongying Zhu, Yi Huang.

**Data curation:** Hongying Zhu, Liurong Cheng.

**Formal analysis:** Liurong Cheng.

**Funding acquisition:** Yi Huang.

**Investigation:** Liurong Cheng.

**Methodology:** Liurong Cheng, Ting Tang.

**Project administration:** Yi Huang.

**Resources:** Ting Tang, Yajuan Ke.

**Software:** Ting Tang, Yajuan Ke.

**Supervision:** Yi Huang.

**Validation:** Yajuan Ke, Duozhi Wu.

**Visualization:** Yajuan Ke, Duozhi Wu.

**Writing – original draft:** Hongying Zhu, Yi Huang.

**Writing – review & editing:** Hongying Zhu, Yi Huang.
